# On “Inlays”

**Published:** 1891-12

**Authors:** W. B. Ames

**Affiliations:** 70 State St., Chicago; New York


NEW YORK, August 7, 1891.

TO THE EDITOR:

DEAR SIR,—I have noticed that in an editorial in the August number of the INTERNATIONAL DENTAL JOURNAL, an attempt is made to establish the date of the introduction into dental practice of the operation termed inlay; that reference is made to a report on Dental Art and Invention made by me to the 1891 meeting of the Illinois State Dental Society, in which inlays were touched upon, and that mention is made of several descriptions of different forms of inlays as appearing in the journals during the latter part of the year previous.

While I am the last person to quibble over the question of priority where it would be so difficult to trace the conception of a method to its original source, I wish to put you in possession of additional data which may help to forestall any complications arising from patents covering the operation.

I was somewhat surprised on reading your editorial that you had not noticed in the transactions of the previous year—viz., 1890—some remarks made by me on the subject of inlays, which remarks were the substance of a paper read before the Odontological Society of Chicago several months previous to the date of the State meeting.

In connection with the gold inlay, with which variety I have had most to do, I can refer you to some matter dating much further than these presentations,—viz., a description in the second edition of Dr. George Evans's "Crown- and Bridge-Work" of the method as practised by Dr. H. A. Parr. This, I might say, dated back with Dr. Parr to December, 1888. When in conversation with Drs.

Parr and Evans, while visiting in their city, I described in detail this operation, which I had in a few instances at that time performed.

From what source I first obtained the idea of filling a gold or platinum matrix with gold solder or other materials, I do not attempt to recall, since this method has no doubt evolved step by step from an origin that would be very difficult of discovery. I remember that at the time of the bringing out, about 1887, of the patented platinum and porcelain inlay by C. H. Land, of Detroit, Mich., I did not recognize anything of an especially novel nature except that he used a furnace of novel construction, and some materials the composition of which was kept secret.

I trust that since the Dental Protective Association has become a firmly-established institution, we are in no danger of being harassed by any ambitious person, who might otherwise attempt to monopolize this valuable operation.

I am yours very sincerely,

W. B. Ames, D.D.S.

70 State St., Chicago.

[The object of the editorial alluded to was to call the attention of the profession to the importance of carefully noting facts as they are presented, and did not claim to be more than approximately correct. The subject of "Inlays" was introduced with the hope that this important and growing process would be traced to its source by some one with more time than the busy editor of this journal. Perhaps no one is better qualified for this labor than the writer of the letter.—Ed.]

